# Professional Ethics

**Published:** 1903-08-15

**Authors:** C. N. Johnson


### PROFESSIONAL ETHICS.

BY C. N. JOHNSON.

Men may talk loftily and loudly about not caring what the profession thinks of them so long as they make the money; but sooner or later, money or no money, there comes to the heart of every man an overwhelming desire to be thought well of by his fellow. He may hide it for a time behind the mask of an artificial complacency, but it will not down. It is human nature. If there is a man who is devoid of this desire, he is far more to be pitied than envied or emulated, and in that man there is not the element of true happiness.

A revelation of this character came to the present writer while he was yet a young man starting out in practice. It so befell that he chanced to meet and talk with an elderly practitioner in another profession who had almost a national reputation for quackery. He was quite generally accounted to be the chiefest exponent of quack methods of his time, and was considered a prince in his way and one who was brilliantly successful along those lines. It was supposed that he had grown wealthy by his methods, and that he cordially despised the "so-called ethics" of professional life. Said he to me: "I suppose that you intend to practice dentistry ethically, do you?" I thought I detected the semblance of a sneer in his remarks, but I had the courage to say to him that such had been my intention from my student days, and that I saw no reason for changing my views. He looked at me a moment with a queer expression, which soon took on a serious turn, and then broke out: "My dear boy, whatever you do, stick to that. It is the only certain road to success in a profession. I should hate to see a young man like you make the deplorable mistake in life that I have made"—only he did not use the word "deplorable." And then he painted such a picture of what his life really was, in contra-distinction to what the world thought it was and what he wished it were, that I never have escaped the impression his recital made upon me.

Let the young man be honor-bright in every relation of life, but particularly let him avoid trafficking with things so sacred as the religious convictions of the community in which he lives. I would rather cope with an open-handed devil any time than trust for one moment the fawning pretense of a sanctimonious hypocrite. Permanent success never yet came from following unworthy methods, and of all of these methods none is more detestable than the one of working the church for professional purposes.

The dentist should early seek to establish a community of interest between the patient and himself, so that their relationship becomes something more than a mere barter of money for professional services. He who developes an abiding friendship between himself and those who come to his office will never lack for patients, and some of the most cherished associations of a lifetime may be made in this way.

